# Bis(cyanamide-κ*N*)[4-(1*H*-imidazol-1-yl)phenol-κ*N* ^3^]bis­(nitrato-κ*O*)copper(II)

**DOI:** 10.1107/S1600536811032399

**Published:** 2011-08-17

**Authors:** Rui-Jin Yu, Bin Deng

**Affiliations:** a College of Science, Northwest A&F University, Yangling, Shaanxi 712100, People’s Republic of China; b Department of Chemistry and Life and Science, Xiangnan University, Chenzhou, Hunan 423000, People’s Republic of China

## Abstract

A pair of linear cyanamide (NCNH_2_) ligands, two monodentate 4-(1*H*-imidazol-1-yl)phenol (*L*) ligands and two nitrate anions link the Cu^II^ atom into a mononuclear unit, [Cu(NO_3_)_2_(C_9_H_8_N_2_O)_2_(NCNH_2_)_2_]. The coordination polyhedron of the Cu atom is an elongated octa­hedron distorted by Jahn–Teller effects. Inter­molecular O—H⋯O, O—H⋯N, N—H⋯O and N—H⋯N hydrogen-bonding inter­actions link these units into a three-dimensional supra­molecular architecture.

## Related literature

For background to related compounds, see: Ferlay *et al.* (1995 ▶); Ribas *et al.* (1999 ▶). For related structures, see: Becker *et al.* (2000 ▶); Berger & Schnick (1994 ▶); Liao & Dronskowski (2006 ▶); Liu *et al.* (2005 ▶); Meyer *et al.* (2000 ▶); Chaudhuri *et al.* (1985 ▶); Tanabe *et al.* (2002 ▶); Yuan *et al.* (2004 ▶, 2007 ▶). 
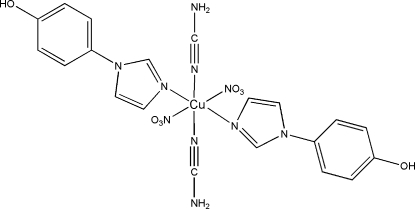


## Experimental

### 

#### Crystal data

[Cu(NO_3_)_2_(C_9_H_8_N_2_O)_2_(CH_2_N_2_)_2_]
*M* *_r_* = 592.00
Triclinic, 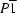

*a* = 8.2235 (7) Å
*b* = 8.7144 (8) Å
*c* = 9.4553 (9) Å
α = 110.808 (1)°
β = 96.696 (2)°
γ = 98.883 (2)°
*V* = 614.92 (10) Å^3^
*Z* = 1
Mo *K*α radiation
μ = 0.96 mm^−1^
*T* = 273 K
0.25 × 0.21 × 0.18 mm

#### Data collection

Bruker SMART CCD area-detector diffractometer
Absorption correction: multi-scan (*SADABS*; Sheldrick, 2002 ▶) *T*_min_ = 0.796, *T*_max_ = 0.847
4951 measured reflections
2396 independent reflections
2229 reflections with *I* > 2σ(*I*)
*R*_int_ = 0.016

#### Refinement

*R*[*F* ^2^ > 2σ(*F* ^2^)] = 0.041
*wR*(*F* ^2^) = 0.105
*S* = 1.07
2396 reflections
178 parameters
H-atom parameters constrained
Δρ_max_ = 0.44 e Å^−3^
Δρ_min_ = −0.18 e Å^−3^

### 

Data collection: *SMART* (Bruker, 1998 ▶); cell refinement: *SAINT-Plus* (Bruker, 1999 ▶); data reduction: *SAINT-Plus*; program(s) used to solve structure: *SHELXS97* (Sheldrick, 2008 ▶); program(s) used to refine structure: *SHELXL97* (Sheldrick, 2008 ▶); molecular graphics: *XP* in *SHELXTL* (Sheldrick, 2008 ▶); software used to prepare material for publication: *SHELXTL*.

## Supplementary Material

Crystal structure: contains datablock(s) I, global. DOI: 10.1107/S1600536811032399/pk2338sup1.cif

Structure factors: contains datablock(s) I. DOI: 10.1107/S1600536811032399/pk2338Isup2.hkl

Additional supplementary materials:  crystallographic information; 3D view; checkCIF report

## Figures and Tables

**Table 1:** Hydrogen-bond geometry (Å, °)

*D*—H⋯*A*	*D*—H	H⋯*A*	*D*⋯*A*	*D*—H⋯*A*
O1—H*O*1⋯O3^i^	0.84	1.88	2.704 (3)	168
O1—H*O*1⋯O4^i^	0.84	2.51	2.970 (3)	115
O1—H*O*1⋯N5^i^	0.84	2.55	3.262 (3)	143
N2—H*N*2*B*⋯O4^ii^	0.86	2.04	2.888 (3)	168
N2—H*N*2*B*⋯O2^ii^	0.86	2.55	3.096 (3)	122
N2—H*N*2*B*⋯N5^ii^	0.86	2.64	3.399 (3)	148
N2—H*N*2*A*⋯O1^iii^	0.86	2.09	2.904 (3)	157
N2—H*N*2*A*⋯O4^iv^	0.86	2.50	3.049 (3)	122

Symmetry codes: (i) 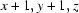; (ii) 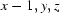; (iii) 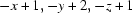; (iv) 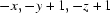.

**Fig. 1.**
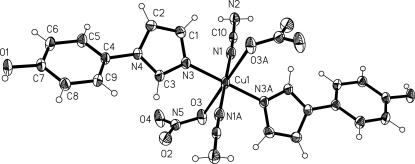
Molecular structure of the title compound. Displacement ellipsoids are potted at the 30% probability level. Atoms with the symmetry code A are related by inversion (-x, 1 - y, -z).

**Fig. 2.**
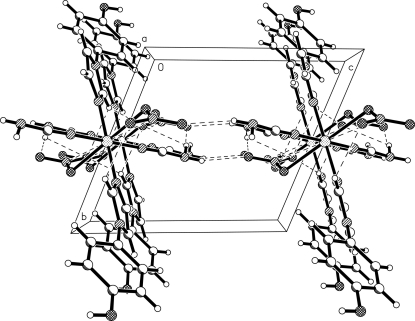
The three-dimensional hydrogen-bonded network in the compound.

## supplementary crystallographic information

### Comment

The design and synthesis of transition-metal coordination compounds with small conjugated molecules and groups, such as cyano, azide, oxalate and nitrido are currently attracting great interest for their diversity of structure and applications in molecule-based magnets (Ferlay *et al.*, 1995; Ribas *et al.*, 1999).  As a potential nitrogen based ligand, cyanamide(NCNH_2_) has been used to prepare a number of alkali metal (Becker *et al.*, 2000), alkaline-earth metal (Berger & Schnick 1994), and rare-earth metal (Liao *et al.*, 2006) salts by different synthetic methods.  Dronskowski and coworkers reported the first and only carbodiimide of a magnetic transition-metal compound in 2005 (Liu *et al.*, 2005). However, to our knowledge, structures of transition-metal cyanamide complexes are limited (Meyer *et al.*, 2000; Chaudhuri *et al.*, 1985; Tanabe *et al.*, 2002; Yuan *et al.*, 2004; Yuan *et al.*, 2007). Since NCNH^-^ is isoelectronic with the azide anion, polymers bridged by NCNH^-^ should also transfer favorable magnetic interactions.  In attempts to synthesize such polymers, the title compound [Cu(*L*)_2_(NCNH_2_)_2_(NO_3_)_2_] was obtained.

The molecular structure of the complex is shown in Fig. 1.  The Cu(II) atom, is located on an inversion center.  The asymmetric unit contains one Cu(II) ion, one *L* ligand, one NCNH_2_, and one nitrate anion.  The Cu(II) atom displays an elongated octahedral geometry with two N atoms from *L* ligand (Cu—N = 1.984 (2) Å), two N atoms from NCNH_2_ (Cu—N = 1.974 (2) Å) and two atoms from NO_3_^-^ (with Cu—O bond length 2.598 (2) Å).  The dihedral angle between the benzene ring and imidazol plane is 40.242 (2) °.

The molecules are assembled into a three-dimensional supramolecular architecture by intermolecular hydrogen-bonding interactions.  The two hydrogen atoms of NCNH_2_ link two neighboring nitrates and one hydroxyl group through N—H···O hydrogen bonds.  The hydroxyl group also connects a nitrate through an O—H···O hydrogen bond.  Each nitrate links two NCNH_2_ and one hydroxyl group from three neighboring units through hydrogen bonds.  These hydrogen bonding interactions extend these units into a three-dimensional molecular architecture (Fig. 2).

### Experimental

To a methanol solution (20 mL) of copper(II) nitrate (0.060 g, 0.25 mmol) and *L* (0.080 g, 0.05 mmol), a water solution (5 ml) of cyanamide (0.020 g, 0.05 mmol) was added slowly with stirring over 30 min. at room temperature. The resulting solution was filtered, and the filtrate was evaporated at room temperature.  After a few days, blue single crystals were obtained (yield: 20%).

### Refinement

H atoms were positioned geometrically and refined as riding atoms, with C—H = 0.93Å and *U*_iso_(H) = 1.2*U*_eq_(C) for aromatic, imidazole H atoms, N—H = 0.86Å and *U*_iso_(H) = 1.2*U*_eq_(C) for amido H atoms, O—H = 0.85Å and *U*_iso_(H) = 1.5*U*_eq_(O) for H atoms of the hydroxyl group.

### Crystal data

**Table:**

[Cu(NO_3_)_2_(C_9_H_8_N_2_O)_2_(CH_2_N_2_)_2_]	*Z* = 1
*M**_r_* = 592.00	*F*(000) = 303
Triclinic, *P*1	*D*_x_ = 1.599 Mg m^−^^3^
Hall symbol: -P 1	Mo *K*α radiation, λ = 0.71073 Å
*a* = 8.2235 (7) Å	Cell parameters from 2396 reflections
*b* = 8.7144 (8) Å	θ = 2.4–26.0°
*c* = 9.4553 (9) Å	µ = 0.96 mm^−^^1^
α = 110.808 (1)°	*T* = 273 K
β = 96.696 (2)°	Block, blue
γ = 98.883 (2)°	0.25 × 0.21 × 0.18 mm
*V* = 614.92 (10) Å^3^	

### Data collection

**Table:**

Bruker SMART CCD area-detector diffractometer	2396 independent reflections
Radiation source: fine-focus sealed tube	2229 reflections with *I* > 2σ(*I*)
graphite	*R*_int_ = 0.016
φ and ω scans	θ_max_ = 26.0°, θ_min_ = 2.4°
Absorption correction: multi-scan (*SADABS*; Sheldrick, 2002)	*h* = −9→10
*T*_min_ = 0.796, *T*_max_ = 0.847	*k* = −10→10
4951 measured reflections	*l* = −11→11

### Refinement

**Table:**

Refinement on *F*^2^	Primary atom site location: structure-invariant direct methods
Least-squares matrix: full	Secondary atom site location: difference Fourier map
*R*[*F*^2^ > 2σ(*F*^2^)] = 0.041	Hydrogen site location: inferred from neighbouring sites
*wR*(*F*^2^) = 0.105	H-atom parameters constrained
*S* = 1.07	*w* = 1/[σ^2^(*F*_o_^2^) + (0.059*P*)^2^ + 0.2239*P*] where *P* = (*F*_o_^2^ + 2*F*_c_^2^)/3
2396 reflections	(Δ/σ)_max_ < 0.001
178 parameters	Δρ_max_ = 0.44 e Å^−^^3^
0 restraints	Δρ_min_ = −0.18 e Å^−^^3^

### Special details

**Table:**

Geometry. All e.s.d.'s (except the e.s.d. in the dihedral angle between two l.s. planes) are estimated using the full covariance matrix. The cell e.s.d.'s are taken into account individually in the estimation of e.s.d.'s in distances, angles and torsion angles; correlations between e.s.d.'s in cell parameters are only used when they are defined by crystal symmetry. An approximate (isotropic) treatment of cell e.s.d.'s is used for estimating e.s.d.'s involving l.s. planes.
Refinement. Refinement of *F*^2^ against ALL reflections. The weighted *R*-factor *wR* and goodness of fit *S* are based on *F*^2^, conventional *R*-factors *R* are based on *F*, with *F* set to zero for negative *F*^2^. The threshold expression of *F*^2^ > σ(*F*^2^) is used only for calculating *R*-factors(gt) *etc*. and is not relevant to the choice of reflections for refinement. *R*-factors based on *F*^2^ are statistically about twice as large as those based on *F*, and *R*- factors based on ALL data will be even larger.

### Fractional atomic coordinates and isotropic or equivalent isotropic displacement parameters (Å^2^)

**Table:**

	*x*	*y*	*z*	*U*_iso_*/*U*_eq_	
Cu1	0.0000	0.5000	0.0000	0.03730 (17)	
O1	1.0200 (2)	1.3124 (2)	0.3638 (2)	0.0522 (5)	
HO1	1.0559	1.3039	0.2822	0.078*	
O3	0.1612 (2)	0.3321 (3)	0.1247 (2)	0.0588 (5)	
O4	0.3655 (3)	0.4115 (4)	0.3168 (3)	0.0738 (7)	
O2	0.4149 (3)	0.4075 (3)	0.0983 (2)	0.0598 (5)	
N1	−0.1364 (3)	0.5376 (3)	0.1629 (3)	0.0465 (5)	
N2	−0.2913 (3)	0.5888 (3)	0.3748 (3)	0.0516 (6)	
HN2B	−0.3876	0.5230	0.3578	0.062*	
HN2A	−0.2331	0.6083	0.4636	0.062*	
N3	0.1547 (3)	0.7184 (2)	0.1239 (2)	0.0390 (5)	
N4	0.3757 (2)	0.9254 (2)	0.2240 (2)	0.0389 (5)	
N5	0.3155 (3)	0.3834 (3)	0.1788 (3)	0.0433 (5)	
C1	0.1064 (3)	0.8598 (3)	0.2122 (3)	0.0473 (6)	
H1	−0.0025	0.8663	0.2274	0.057*	
C2	0.2407 (3)	0.9884 (3)	0.2740 (3)	0.0484 (7)	
H2	0.2417	1.0981	0.3378	0.058*	
C3	0.3189 (3)	0.7628 (3)	0.1334 (3)	0.0392 (6)	
H3	0.3853	0.6910	0.0841	0.047*	
C4	0.5449 (3)	1.0196 (3)	0.2591 (3)	0.0372 (5)	
C5	0.6081 (3)	1.1383 (3)	0.4066 (3)	0.0459 (6)	
H5	0.5432	1.1533	0.4828	0.055*	
C6	0.7685 (3)	1.2339 (3)	0.4393 (3)	0.0460 (6)	
H6	0.8122	1.3130	0.5381	0.055*	
C7	0.8639 (3)	1.2124 (3)	0.3258 (3)	0.0393 (6)	
C8	0.8007 (3)	1.0926 (3)	0.1790 (3)	0.0427 (6)	
H8	0.8656	1.0774	0.1028	0.051*	
C9	0.6406 (3)	0.9957 (3)	0.1462 (3)	0.0415 (6)	
H9	0.5980	0.9146	0.0481	0.050*	
C10	−0.2100 (3)	0.5568 (3)	0.2602 (3)	0.0387 (6)	

### Atomic displacement parameters (Å^2^)

**Table:**

	*U*^11^	*U*^22^	*U*^33^	*U*^12^	*U*^13^	*U*^23^
Cu1	0.0265 (2)	0.0408 (3)	0.0357 (3)	−0.00682 (16)	0.00593 (17)	0.00971 (18)
O1	0.0305 (10)	0.0649 (12)	0.0477 (11)	−0.0127 (8)	0.0005 (8)	0.0169 (9)
O3	0.0357 (11)	0.0773 (14)	0.0555 (12)	−0.0089 (9)	0.0010 (9)	0.0269 (10)
O4	0.0382 (12)	0.125 (2)	0.0533 (13)	−0.0089 (12)	0.0036 (10)	0.0399 (13)
O2	0.0486 (12)	0.0711 (13)	0.0574 (13)	0.0021 (10)	0.0240 (10)	0.0215 (10)
N1	0.0330 (12)	0.0554 (13)	0.0397 (12)	−0.0069 (10)	0.0084 (10)	0.0108 (10)
N2	0.0368 (12)	0.0724 (15)	0.0368 (12)	0.0016 (11)	0.0091 (10)	0.0138 (11)
N3	0.0290 (11)	0.0387 (11)	0.0431 (12)	−0.0023 (8)	0.0051 (9)	0.0127 (9)
N4	0.0281 (11)	0.0352 (10)	0.0461 (12)	−0.0018 (8)	0.0040 (9)	0.0111 (9)
N5	0.0352 (12)	0.0428 (11)	0.0474 (13)	0.0025 (9)	0.0101 (10)	0.0132 (10)
C1	0.0298 (13)	0.0472 (14)	0.0621 (17)	0.0050 (11)	0.0115 (12)	0.0176 (13)
C2	0.0367 (14)	0.0362 (13)	0.0633 (18)	0.0045 (10)	0.0115 (13)	0.0090 (12)
C3	0.0291 (12)	0.0369 (12)	0.0439 (14)	−0.0008 (10)	0.0059 (10)	0.0098 (10)
C4	0.0279 (12)	0.0341 (11)	0.0448 (14)	−0.0013 (9)	0.0027 (10)	0.0139 (10)
C5	0.0369 (14)	0.0490 (14)	0.0430 (14)	−0.0049 (11)	0.0085 (11)	0.0125 (11)
C6	0.0392 (14)	0.0472 (14)	0.0378 (14)	−0.0070 (11)	0.0001 (11)	0.0086 (11)
C7	0.0279 (12)	0.0402 (12)	0.0461 (14)	−0.0017 (10)	0.0002 (10)	0.0175 (11)
C8	0.0338 (13)	0.0488 (14)	0.0415 (14)	0.0039 (11)	0.0086 (11)	0.0139 (11)
C9	0.0342 (13)	0.0382 (12)	0.0405 (14)	−0.0001 (10)	−0.0006 (11)	0.0069 (10)
C10	0.0281 (12)	0.0408 (13)	0.0386 (14)	−0.0028 (10)	−0.0008 (11)	0.0114 (10)

### Geometric parameters (Å, °)

**Table:**

Cu1—N1	1.974 (2)	N4—C2	1.372 (3)
Cu1—N1^i^	1.974 (2)	N4—C4	1.438 (3)
Cu1—N3	1.9837 (19)	C1—C2	1.349 (4)
Cu1—N3^i^	1.9837 (19)	C1—H1	0.9300
O1—C7	1.365 (3)	C2—H2	0.9300
O1—HO1	0.8409	C3—H3	0.9300
O3—N5	1.259 (3)	C4—C9	1.375 (4)
O4—N5	1.244 (3)	C4—C5	1.388 (4)
O2—N5	1.224 (3)	C5—C6	1.383 (4)
N1—C10	1.136 (3)	C5—H5	0.9300
N2—C10	1.308 (3)	C6—C7	1.378 (4)
N2—HN2B	0.8638	C6—H6	0.9300
N2—HN2A	0.8613	C7—C8	1.386 (4)
N3—C3	1.329 (3)	C8—C9	1.385 (3)
N3—C1	1.368 (3)	C8—H8	0.9300
N4—C3	1.342 (3)	C9—H9	0.9300
			
N1—Cu1—N1^i^	180.00 (14)	C1—C2—H2	126.8
N1—Cu1—N3	89.82 (9)	N4—C2—H2	126.8
N1^i^—Cu1—N3	90.18 (8)	N3—C3—N4	110.7 (2)
N1—Cu1—N3^i^	90.18 (9)	N3—C3—H3	124.7
N1^i^—Cu1—N3^i^	89.82 (8)	N4—C3—H3	124.7
N3—Cu1—N3^i^	180.0	C9—C4—C5	120.6 (2)
C7—O1—HO1	108.5	C9—C4—N4	120.2 (2)
C10—N1—Cu1	177.0 (2)	C5—C4—N4	119.1 (2)
C10—N2—HN2B	116.0	C6—C5—C4	119.4 (3)
C10—N2—HN2A	115.8	C6—C5—H5	120.3
HN2B—N2—HN2A	112.2	C4—C5—H5	120.3
C3—N3—C1	106.0 (2)	C7—C6—C5	120.2 (2)
C3—N3—Cu1	129.22 (18)	C7—C6—H6	119.9
C1—N3—Cu1	124.71 (17)	C5—C6—H6	119.9
C3—N4—C2	107.3 (2)	O1—C7—C6	117.7 (2)
C3—N4—C4	127.0 (2)	O1—C7—C8	122.1 (2)
C2—N4—C4	125.7 (2)	C6—C7—C8	120.2 (2)
O2—N5—O4	120.4 (2)	C9—C8—C7	119.7 (2)
O2—N5—O3	120.9 (2)	C9—C8—H8	120.1
O4—N5—O3	118.8 (2)	C7—C8—H8	120.1
C2—C1—N3	109.6 (2)	C4—C9—C8	119.8 (2)
C2—C1—H1	125.2	C4—C9—H9	120.1
N3—C1—H1	125.2	C8—C9—H9	120.1
C1—C2—N4	106.5 (2)	N1—C10—N2	176.6 (3)

Symmetry codes: (i) −*x*, −*y*+1, −*z*.

### Hydrogen-bond geometry (Å, °)

**Table:**

*D*—H···*A*	*D*—H	H···*A*	*D*···*A*	*D*—H···*A*
O1—HO1···O3^ii^	0.84	1.88	2.704 (3)	168.
O1—HO1···O4^ii^	0.84	2.51	2.970 (3)	115.
O1—HO1···N5^ii^	0.84	2.55	3.262 (3)	143.
N2—HN2B···O4^iii^	0.86	2.04	2.888 (3)	168.
N2—HN2B···O2^iii^	0.86	2.55	3.096 (3)	122.
N2—HN2B···N5^iii^	0.86	2.64	3.399 (3)	148.
N2—HN2A···O1^iv^	0.86	2.09	2.904 (3)	157.
N2—HN2A···O4^v^	0.86	2.50	3.049 (3)	122.

Symmetry codes: (ii) *x*+1, *y*+1, *z*; (iii) *x*−1, *y*, *z*; (iv) −*x*+1, −*y*+2, −*z*+1; (v) −*x*, −*y*+1, −*z*+1.
